# Unilateral Epidural Blockade for Lower Limb Fracture Surgery: Parasagittal Epidural Versus Midline Epidural Anesthesia

**DOI:** 10.29252/beat-070210

**Published:** 2019-04

**Authors:** Masoud Hashemi, Payman Dadkhah, Mehrdad Taheri, Sirous Momenzadeh, Tahereh Parsa, Behnam Hosseini, Mohammadreza Abbasian

**Affiliations:** 1 *Department of Anesthesiology and Pain, Akhtar Hospital, Shahid Beheshti University of Medical Sciences, Tehran, Iran*; 2 *Department of Anesthesiology and Pain, Labbafi Nejad Hospital, Shahid Beheshti University of Medical Sciences, Tehran, Iran*; 3 *Department of Anesthesiology and Pain, Imam-Hossein Hospital, Shahid Beheshti University of Medical Sciences, Tehran, Iran*; 4 *Department of Anesthesiology and Pain, Masih-Daneshvari Hospital, Shahid Beheshti University of Medical Sciences, Tehran, Iran*; 5 *Department of Orthopedic Surgery, Akhtar Hospital, Shahid Beheshti University of Medical Sciences, Tehran, Iran*

**Keywords:** Anesthesia, Epidural, Interlaminar approach, Parasagittal approach motor block, Sensory block, Hemodynamics stability, Success rate

## Abstract

**Objective:**

To compare the efficacy of parasagittal interlaminar (PIL) and midline interlaminar (MIL) approaches for epidural block in patients with lower limb orthopedic surgery.

**Methods:**

This double-blind randomized clinical trial was performed on 40 patients undergoing tibial shaft fracture surgery. In PIL group, an 18-gauge, 3.5 inch, Tuohy needle was placed at the level of L2-3 or L3-4 intervertebral spaces and pushed forward in a posteroanterior (PA) direction vertical to the body surface. After determining the most lateral place for needle arrival in an anteroposterior (AP) view, needle was pushed forward into the epidural space. For the MIL group, needle was pushed forward from the midline interspinous space with the same method. After confirmation of needle position, 1 mL of contrast was injected to confirm the epidural space distribution and then 15 ml lidocaine 2% was injected. The sensory and motor block level, onset, duration, heart rate (HR), mean arterial pressure (MAP), and arterial oxygen saturation (SPO2), and success rate were recorded.

**Results:**

Mean patients’ baseline characteristics showed no statistically significant difference between the two groups.*p*>0.05). Outcome measures were statistically different and significantly higher in PIL group (p-values for sensory block level <0.001, motor block level <0.001, duration of sensory block: <0.001 and duration of motor block <0.001 and success rate: <0.001). Hemodynamic variables didn’t show statistically significant difference between the two groups (p-values for Systolic pressure: 0.997, diastolic pressure:0.579, MAP:0.585, HR:0.710).

**Conclusion:**

Epidural anesthesia with parasagittal interlaminar approach provide deep motor block, high sensory level block, and hemodynamic stability.

**Clinical trial registry:**

IRCT2017041615515N2

## Introduction

In orthopedic surgical patients, it is important and necessary to take the intra- and postoperative management issues into account. The early mobility and rehabilitation after surgery with minimal pain and discomfort are among the most desirable features of modern surgical procedures [1-3]. To appropriately manage anesthesia, it is necessary to have proper knowledge about possible complications, adequate preparation before surgery with minimal physiological disturbances and also adverse effects [4]. According to the findings of recent studies, progress in anesthesia and analgesia can influence the postoperative outcomes [5-7]. Nowadays, regional anesthesia techniques are increasingly applied in orthopedic surgeries [8]. Compared to general anesthesia, regional anesthesia is associated with more benefits such as the intra-operative consciousness of patient, spontaneous respiration, defensive airway reflexes and post-operative analgesic preparation. 

Epidural anesthesia and analgesia make it possible to decrease or eliminate the physiological stress responses before surgery and thereby, reduce surgery-related complications and improve the outcome [5-7]. Epidural anesthesia is an anesthetic technique that is commonly used in lower limb surgery and provides patients with more preoperative comfort, compared to the general anesthesia. Ventral extensions of the contrast with insertion of needles in the most lateral part of the interlaminar space have been reported to be about 100% [9, 10]. The parasagittal interalaminar route can be associated with good ventral epidural spread with fewer complications compared to the transforaminal route [11]. In these investigations, only the schema of contrast spread has been studied and the clinical importance of the results in lower limb surgery have not been assessed. There is no report on the controlled randomized trials in the literature comparing the parasagittal interlaminar (PIL) and midline interlaminar (MIL) routes for clinical outcomes in patients undergoing lower limb orthopedic surgeries. Therefore, the present study was conducted to compare the clinical efficacies of the PIL and MIL approaches in patients with lower limb orthopedic surgery. We supposed that compared to MIL approach, the PIL method may result in better clinical outcomes for ventral epidural spread of drugs. 

## Materials and Methods

 *Study population*

  This double-blind randomized clinical trial was performed on 40 patients in a university hospital during 2016. Study method was approved by institution review board of ethics and study protocol was registered in Iranian Registry of Clinical Trials under ID of IRCT2017041615515N2. Written informed consent forms were signed by all participants. Study population consisted of patients presented with tibial shaft fracture referring to our university hospital and scheduled for tibial shaft fracture surgery under regional anesthesia. Inclusion criteria was age ≥18 years, ASA physical status class I-II. Pregnant women, sepsis, emergency surgeries, infection at the site of injection, coagulopathy or other bleeding diathesis, spinal deformity, diabetes, significant systemic disease and neuropathy, history of surgery on the lumbar spine, facet joint arthropathy, cauda equina syndrome and other unstable neurologic deficits, cases with allergy to contrast or lidocaine were excluded from the study.

*Randomization and intervention*

 Sampling was done using census method and participants were randomly subdivided into MIL (n=20) and PIL (n=20) groups through block randomization. For all patients, standard monitoring (non –invasive arterial blood pressure, pulse oximetry and electrocardiography) was applied on arrival to the operating room. Procedural sedation was achieved using 1mg/ml of intravenous midazolam. In the PIL group, patients were placed in the sitting position. After sterilization and preparation of the site, the skin was anesthetized by subcutaneous injection of 3ml 1% lidocaine. Next, an 18-gauge, 3.5 inch, Tuohy needle was placed at the level of L2-3 or L3-4 intervertebral spaces and pushed forward in a posterior to anterior direction vertical to the body surface. After determining the most lateral place for needle arrival in a fluoroscopic anterior-posterior (AP) view, the needle was pushed forward into the epidural space of the operated side, using the loss-of-resistance technique and this parasagittal direction of the needle was maintained throughout the whole procedure. Bevel side was placed toward lateral [12]. For the MIL group, the needle was pushed forward from the midline interspinous space with the same method. In none of patients in the two groups the catheter was pushed forward. Once the needle was placed in proper position, and after negative aspiration for cerebrospinal fluid and blood, 1 mL of the contrast dye (OMNIPAQUE ™, GE Healthcare, UK) was injected to confirm the epidural space distribution in the AP view. After epidural space confirmation, 15 ml of lidocaine 2% was injected. Patients were placed in supine position in order to help create complete sensory and motor blocks. The sensory block level was controlled based on pinprick test, assessed by a verbal rating scale from 100% (normal sensation) to 0 (no sensation). Motor block level was evaluated using Bromage score [13] as: I Nil (Free movement of feet and legs), II partial (just being able to flex the knee with the free movement of feet), III almost complete (inability to bend the knee, while being able to freely move the feet),IV complete (inability to move the legs or feet). 

The sensory and motor block onset time was defined as the time between the end of the last injection and complete absence of pinprick response and complete paralysis (Bromage score = IV) in all nerve distributions. Block onset and levels were recorded. In the case of block failure of any nerve distribution, the patient was excluded from the study. Surgery was initiated after establishing sensory and motor blocks at the surgery site. Sensory and motor block were checked continuously every 10 minutes following the end of operation. 

*Outcome measures*

  Sensory and motor block duration were recorded for both sides. The duration of sensory block was defined as the time interval between complete sensory block (complete absence of pinprick response) and the first postoperative pain. The duration of motor block was defined as the time interval between complete paralysis (Bromage score = IV) and complete recovery (Bromage score = I). Hemodynamic parameters (HR, MAP, and SPO2) were recorded right before anesthesia and then every 10 minutes until the end of surgery. Hypotension was considered to be significant, if decline from the baseline was more than 30% and was treated with ephedrine and IV fluids. Bradycardia, was considered to be significant, if the heart rate was less than 50 bpm which was treated with atropine. In case of bladder dysfunction, a urinary catheter insertion was considered. The success rate was defined based on the number of patients achieving complete motor block intraoperatively. Complications (nausea, vomiting, itching, headache, hypotension, bradycardia, bleeding and urinary retention) were recorded for both operated and non-operated sides. After the end of surgery, patients were transferred to PACU and observed for side effects such as nausea, vomiting, and urinary retention. Patients were discharged from PACU after stabilizing vital signs and normal voiding. 

*Statistical analysis*

  Using two sample mean comparison test, study sample size was arrived at 20 for each group, assuming an α-error of 0.05, power of 80%, and drop-out rate of 10%. Data analysis was performed using SPSS 19.0 software (Chicago, Illinois, USA). Chi-Square and Independent-Sample-T tests as well as Man-Whitney U test were used after determination of parametric distribution according to Kolmogorov-Smirnov test, and measurement outcomes were compared through the Repeated-Measurement ANOVA.  *P*-value less than 0.05 was considered to be statistically significant.

## Results

**Fig. 1:**
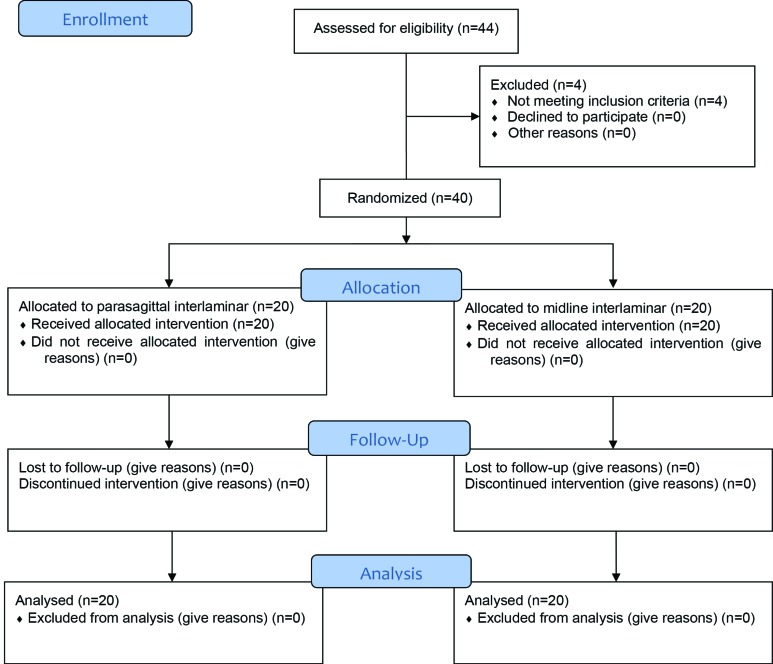
CONSORT flow diagram of the study

Overall we screened 44 patients for eligibility and 40 were finally randomized and all the patients finished the study (Figure 1). The patients’ mean age, sex, height, weight, BMI, ASA physical status class, and duration of surgery were the same in two groups and no statistically significant difference between the two groups was noted (Table 1). Sensory and motor block level (Bromage score: IV) was significantly higher in the PIL group (*p*=0.037). The onset of sensory and motor block was 17.8±1 min in the PIL group and 18±0.5 min in the MIL group (*p*=0.445). Duration of sensory block time was significantly higher in the PIL group, in comparison with the MIL one (*p*=0.0001). Duration of motor block was significantly higher in the PIL group (*p*=0.002). The success rate was significantly higher in the PIL group (95%) compared to that in the MIL group (70%) (*p*=0.037). None of patients developed any complication. Maximum sensory block level reached in the operated side was between T12 and T8 in the PIL group and between T10 and T9 in the MIL group. There was a significant difference between the two groups (*p*=0.0001) (Table 2). 

**Table 1:** Demographic and clinical data of the patients under investigation

	**PIL group** **(n=20)**	**MIL group** **(n=20)**	***P*** ** value**
**Age (yr.) **	30.3±3.8	28.2±4.6	0.109
**Weight (kg)**	75.4±15.4	78.7±13.5	0.270
**Height (cm)**	167.1±7.5	165.2±9.6	0.550
**BMI (kg/m2)**	26.9±4.5	28.9±4.9	0.283
**Duration of surgery**	120.8±1.6	120.0±0.9	0.192
**Sex **			
**Male** **Female**	12 (60%)8 (40%)	14 (70%)6 (30%)	0.507
**ASA class **			
**I** **II**	15 (75%)5 (25%)	12 (60%)8 (40%)	0.311
**Block success rate (Bromage score)**			
**III** **IV **	1 (5%)19 (95%)	6 (30%)14 (70%)	0.037
**Duration of sensory block (min)**	158.4±1.4	156.6±1.04	0.0001
**Duration of motor block (min)**	127.3±3.1	123.9±3.6	0.002

**Table 2:** Maximum sensory block level in the operated side

	**T12**	**T10**	**T9**	**T8**	***P*** ** value**
**PIL **	12 (60%)	-	1 (5%)	7 (35%)	0.0001
**MIL**	-	13 (65%)	7 (35%)	-	

**Fig. 2:**
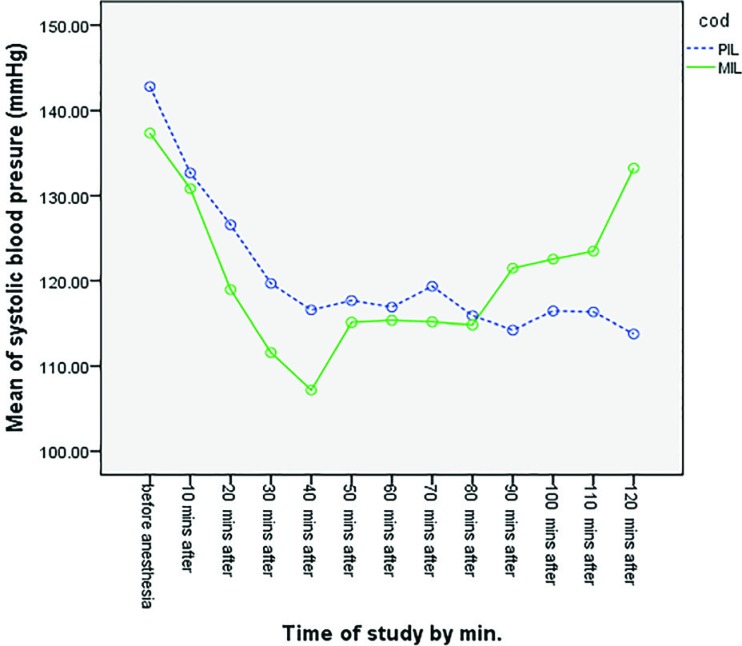
Comparison of mean systolic blood pressure in two groups at different time points (*p*=0.997)

**Fig. 3:**
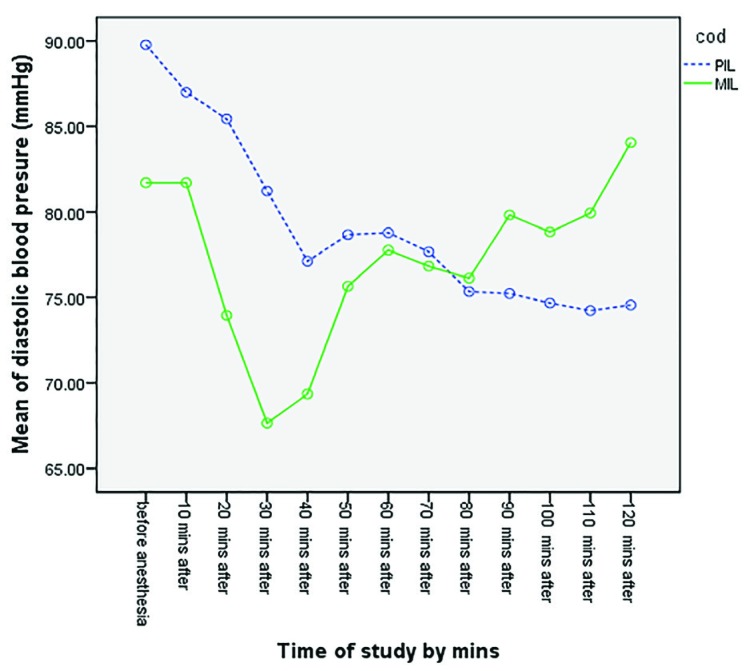
Comparison of mean diastolic blood pressure in two groups at different time points (*p*=0.579)

**Fig. 4:**
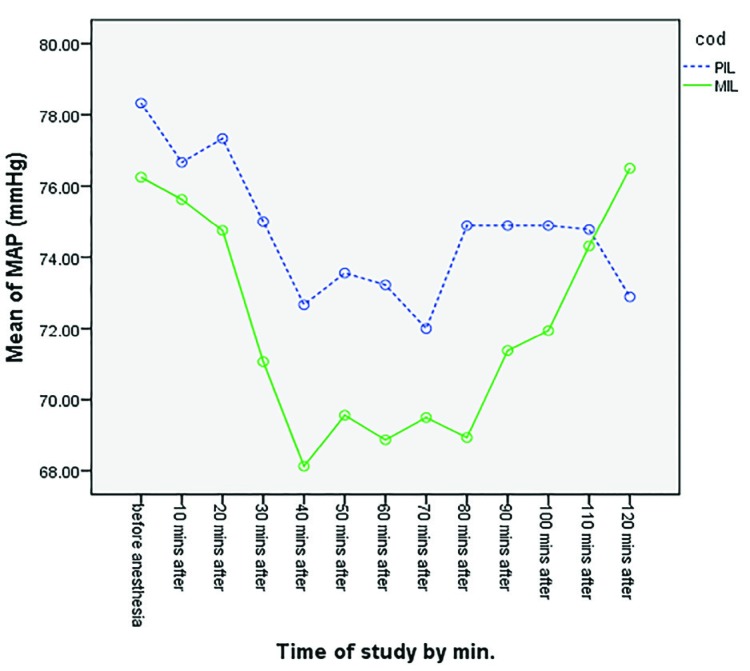
Comparison of mean MAP in two groups at different time points (*p*=0.585)

**Fig. 5:**
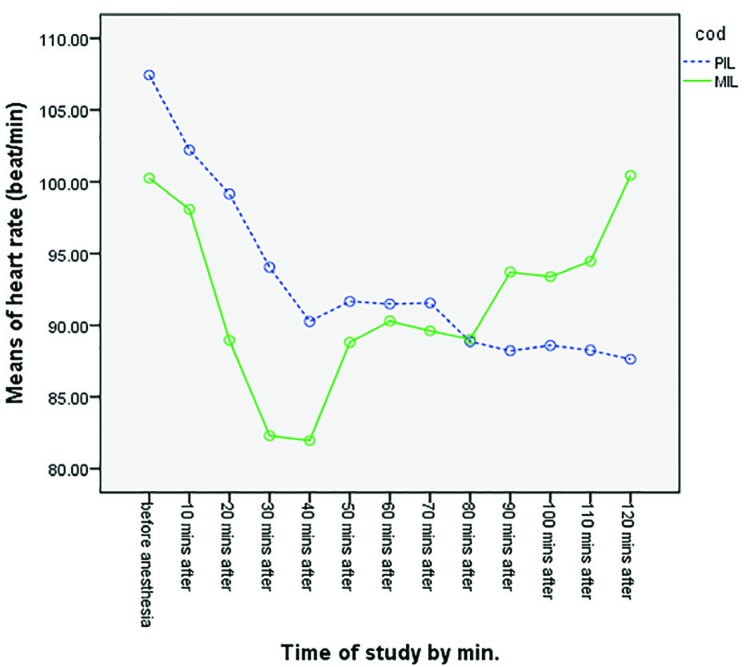
Comparison of mean HR in two groups at different time points (*p*=0.710)

Higher levels of the maximum sensory block were observed in the PIL Group, in comparison with the MIL group. The maximum sensory block level reached in the non-operated side was between L3 and T9 in the PIL group and between T10 and T9 in the MIL group. There was a significant difference between two groups (*p*=0.0001) (Table 3). Maximum sensory block was at lower levels in the PIL group, compared to the MIL group.  Changes in the systolic blood pressure at different time points during the surgery in both studied groups have been demonstrated in Figure 2. No statistically significant difference was observed between the two groups (*p*=0.997). Changes in the diastolic blood pressure at different time points during the surgery in both studied groups have been demonstrated in Figure 3. No statistically significant difference was observed between the two groups (*p*=0.579). Changes in MAP at different times during the surgery in both studied groups have been demonstrated in Figure 4. No statistically significant difference was observed between the two groups (*p*=0.585). Changes in the heart rate at different times during the surgery in both studied groups have been demonstrated in Figure 5. No statistically significant difference was observed between the two groups (*p*=0.710).

**Table 3:** Maximum sensory block level in the non-operated side

	**L3**	**L2**	**L1**	**T12**	**T10**	**T9**	***P*** ** value**
**PIL **	6 (30%)	5 (25%)	7 (35%)	2 (10%)	-	-	0.0001
**MIL**	-	-	-	-	16 (80%)	4 (20%)	

## Discussion

Unilateral epidural block is usually used to manage chronic pains [14]. However, in the present study, we used this method for surgery. A one-side sensory and/or motor block may be created after placement of an epidural catheter. Although many case studies have reported various adverse events in different clinical settings [15-18], the occurrence of unilateral epidural block in the operative side may be beneficial in orthopedic patients with unilateral surgery in the lower limb. However, only a few randomized clinical trials have examined the clinical outcomes in unilateral epidural anesthesia [19, 20]. The present study is the first report comparing the effects of PIL and MIL approaches in the epidural block technique without catheter placement after a single injection on the epidural block distribution for intraoperative anesthesia. Fukushige *et al*. have proposed that the dorsomedian connective tissue band might act as a physical barrier to dissemination of the local anesthetic agent, leading to a unilateral epidural block [21]. In contrast, Hogan *et al*. have reported that in unilateral epidural block, catheter position is the dominant factor (compared to the anatomic barriers) [22]. Our results confirmed the supposition of Fukushige *et al*., [21] regarding the role of tissue barriers in establishing unilateral epidural block.  In our study, the success rate was significantly higher in the PIL group (95%), compared to the MIL group (70%), in agreement with the results of a previous investigation by Hashemi *et al*., [12], in which the parasagittal approach led to a more successful drug delivery to the ventral epidural space compared to the midline approach (with 75% and 25% success rates, respectively). In another study, Ghai *et al*., [11] have reported a significantly higher ventral epidural spread of the contrast in the Parasagittal Interlaminar (PIL) group (89.7%) compared to that in the midline interlaminar (MIL) group (31.7%). With recent advances in the anatomy of epidural space, the accurate anatomical diffusion of drugs into the epidural space has attracted more scientific interest. Anatomical and physical factors have been considered to be effective in the ventral spread of drugs [12].

Some authors have reported that patient position and gravity can also affect the unilateral blocks [18, 23-25].Dispensation of drugs closed to the operated side can provide many benefits, especially in orthopedic patients undergoing unilateral surgery. Limiting the epidural block to the surgical site will come with a lot of advantages, similar to the case of unilateral spinal anesthesia [25]. In our study, maximum sensory block level in the operated side was between T12-T8 in the PIL group and T10-T9 in the MIL group. Moreover, the maximum sensory block level in the non-operated side was between L3-T12 in the PIL group and between T10-T9 in the MIL group. These results were in agreement with the findings of Borghi *et al*., [17], Dauri *et al*., [23] and Boyacı *et al*., [19], indicating that the rate of expansion of cold sensitivity and loss of touch sensation in the non-operated side was lower in the PIL group. 

In our study, the time durations of sensory and motor blocks were significantly different, being higher in the PIL group. However, no significant difference in the preparation time for surgery was observed between the two groups. These findings were in consistence with the results reported by Borghi *et al*.,  [17] and Boyacı *et al*., [19]. In the present research, changes of hemodynamic parameters in the two groups after anesthesia and during the surgery were not significantly different, consistent with the findings of other studies indicating that hemodynamic are less altered in the unilateral epidural anesthesia [22]. One of the major drawbacks of the present study is small number of sample size and single-center populations. Further multi-central studies with larger sample size are recommended for future studies.

In conclusion, epidural anesthesia with parasagittal interlaminar approach in patients undergoing one–sided lower extremity operations can be used as a successful alternative to other anesthesia techniques in lower extremity surgery, providing deep motor block, high sensory level block, and hemodynamic stability. 

## Conflicts of interests:

The authors declare no conflicts of interest 

## Funding:

 The authors have no sources of funding to declare for this manuscript.
